# Breaking the Cycle: How Coping and Flexibility Disrupt the Link Between Kinesiophobia and Rumination in Athletes

**DOI:** 10.3390/bs15091271

**Published:** 2025-09-17

**Authors:** Osman Pepe, Mehmet Behzat Turan, İbrahim Dalbudak, Berat Koçyiğit, Gül Bahar Bayıroğlu, Melih Balyan, Olcay Mülazımoğlu, Sevim Kır

**Affiliations:** 1 Faculty of Sports Sciences, Süleyman Demirel University, Isparta 32260, Türkiye; osmanpepe@sdu.edu.tr (O.P.); beratkocyigit@sdu.edu.tr (B.K.); gulbayiroglu@sdu.edu.tr (G.B.B.); 2 Faculty of Sports Sciences, Erciyes University, Kayseri 38280, Türkiye; 3 Faculty of Sports Sciences, Uşak University, Uşak 64000, Türkiye; ibrahim.dalbudak@usak.edu.tr; 4 Faculty of Sport Sciences, Ege University, Izmir 35040, Türkiye; melih.balyan@ege.edu.tr; 5 Faculty of Sports Sciences, Mugla Sıtkı Koçman University, Muğla 48000, Türkiye; olcaymulazimoglu@mu.edu.tr; 6 Institute of Health Sciences, Erciyes University, Kayseri 38280, Türkiye; ksevim38@outlook.com

**Keywords:** kinesiophobia, rumination, psychological flexibility, coping responses, athlete, sport psychology

## Abstract

Background: This study aims to examine the relationship between Kinesiophobia and Rumination in athletes, and to reveal the mediating roles of Coping Responses and Psychological Flexibility in this relationship. Methods: Three hundred ninety licensed athletes, including 225 females and 165 males, voluntarily participated in the study. Participants were selected through simple random sampling from various sports clubs across Turkey. During the data collection, participants were contacted online. They completed the personal ınformation form, the Tampa Scale of Kinesiophobia, the Sport Competition Rumination Scale, the Psychological Flexibility Scale, and the Coping Responses Inventory. IBM SPSS 26 and PROCESS Macro Model 4.0 were used for data analysis. In addition to descriptive statistics, Pearson correlation, linear regression, and mediation analyses were conducted. The adequacy of the sample size was evaluated using G*Power (v 3.1). The Bootstrap method with 5000 resamples and a 95% confidence interval was applied in the mediation analysis. Results: The study’s findings indicated that kinesiophobia significantly predicted levels of rumination among athletes, and that both psychological flexibility and coping responses partially mediated this relationship. Although both variables were functional in reducing ruminative thinking, coping responses demonstrated a more potent effect. The results suggest that the tendency to avoid movement affects physical and cognitive processes. Conclusions: It was concluded that psychological flexibility and coping responses are protective factors in reducing repetitive negative thought patterns in athletes. In this regard, it is recommended that holistic intervention programs aimed at enhancing psychological resilience be developed to support mental health and athletic performance.

## 1. Introduction

Sport refers to activities in which individuals participate to support their mental, emotional, and physical health while promoting unity and solidarity (Eroğlu & Ersoy, 2019). However, structural impairments and functional losses that may occur during sports activities can lead to sports injuries due to the effects of energy transfer (B. Kılıç et al., 2014; Karayol & Yavuz Eroğlu, 2020). Injuries during physical activity may result from imbalances in the musculoskeletal system, mechanical loading, and the combination of genetic factors (Joyner, 2019; Varillas Delgado et al., 2023).

The factors that increase the risk of injury in athletes can be evaluated across a broad range, including both individual characteristics (e.g., age, gender, physical fitness, history of previous injuries) and environmental conditions (e.g., type of sport, quality of equipment, field conditions, training routine) (Koku, 2013; Budak et al., 2020). Therefore, in every sport, it is inevitable that individuals face various risks ranging from minor injuries to severe damage (Koşar et al., 2006). Injuries experienced by athletes lead not only to physical but also to psychological consequences.

Following an injury, athletes may experience psychological responses such as fear of pain and anxiety about reinjury, which can lead to avoidance of movement and a consequent reduction in physical capacity. This situation may result in Kinesiophobia, which is defined as an excessive and irrational movement avoidance response (De Vroey et al., 2020; Tang et al., 2025). Kinesiophobia may negatively affect not only an athlete’s physical performance but also their self-efficacy perception and overall psychological well-being. Kinesiophobia, while referring to different concepts across cultures and disciplines, is generally defined as an “excessive and disproportionate avoidance response to movement” (Vlaeyen & Linton, 2000).

However, although kinesiophobia is a common issue observed in athletes after injury, for professional athletes, it is often not possible to avoid movement for a prolonged period. Due to the nature of professional sports, injuries are considered one of the most remarkable career-ending threats, and athletes must regain movement to maintain their performance (Ambegaonkar et al., 2024; Kızılay & Burkay, 2023). Therefore, the applicability of the concept of kinesiophobia to professional athletes is debatable, since this group cannot entirely refrain from movement. Thus, unlike in ordinary individuals, where it is manifested through “movement avoidance,” in professional athletes, kinesiophobia can be understood as a fear and mental burden experienced within the obligation to continue participating in sports (Bingöl et al., 2025). Indeed, some studies have reported that amateur athletes struggle more in coping with kinesiophobia, whereas professional athletes focus more on the injury itself and the accompanying mental burden (Ambegaonkar et al., 2024; Kızılay & Burkay, 2023).

In this process, even if actual avoidance of movement is not possible (particularly during rehabilitation), the mental processing of fear is inevitable (Ambegaonkar et al., 2024). In the post-injury period, athletes may develop ruminative thought patterns involving constant mental focus on past negative experiences, which can weaken their coping mechanisms with stress (Karabay et al., 2023). Mainly, emotion-focused and meaning-seeking ruminative tendencies may hinder problem-solving-oriented coping and instead promote emotion-focused coping strategies, thereby negatively affecting performance (Chen et al., 2025). Therefore, managing such mental processes is critical for maintaining athletic performance and supporting safe recovery and return to play.

In order to ensure an early and safe return to sport, rehabilitation protocols for the recovery process must be implemented meticulously (Kocahan, 2025). However, physical recovery alone is insufficient; the athlete’s psychological processes must be closely monitored during this period. In this context, the levels of Coping Responses and Psychological Flexibility exhibited by athletes emerge as critical variables that can directly affect the recovery process and decisions regarding return to sport.

### 1.1. Kinesiophobia

Kinesiophobia delays recovery, complicates return to sport, and adversely affects muscle activity and motor strategies (De Oliveira Silva et al., 2019; Goldberg et al., 2018). The fear avoidance model, introduced by Lethem et al. (1983), provides a fundamental framework for understanding the psychological mechanisms of Kinesiophobia (Sharif-Nia et al., 2025; L. Li et al., 2023). According to this model, individuals develop a fear of pain following an injury or painful experience. This fear triggers avoidance behaviors toward physical activities that are perceived as potentially causing pain or re-injury. While this behavior may initially appear to be a protective strategy in the short term, it ultimately leads to physical inactivity, muscle weakness, functional impairments, and chronic fear of movement. Consequently, as the individual continues to limit movement due to fear of re-injury, a vicious cycle develops physiologically and psychologically.

The definition proposed by Kori et al. (1990) supports this view, indicating that feelings of helplessness in response to injury increase the fear of re-injury among athletes. Knapik et al. (2019) define Kinesiophobia as an internal barrier inhibiting an individual’s physical activity participation (Kluszczyńska et al., 2021). This psychological barrier may diminish athletes’ motivation to move, prolonging the return to sport process (Sevim, 2018).

However, rehabilitation processes often focus predominantly on physiological recovery, whereas psychological and behavioral effects are frequently overlooked (Podlog et al., 2014). However, factors such as stress, anxiety, low self-esteem, and depression observed in athletes may directly affect the recovery process (Kluszczyńska et al., 2021; Bayram, 2020). At this point, a detailed examination of the cognitive processes accompanying Kinesiophobia is of great importance in understanding how negative thought patterns and mental fixations that emerge after injury affect the healing process. In particular, athletes’ avoidance of physical activity due to fear of re-injury can adversely influence both their motor strategies and muscle activity. Similarly, the literature reports that kinesiophobia negatively affects the rehabilitation process and return to play. However, long-term avoidance does not seem feasible due to the pressure on professional athletes to return (Hsu et al., 2017). Moreover, fewer than 50% of athletes can return to their pre-injury activity levels, demonstrating that the only option at the professional level is not complete avoidance of movement but its controlled regaining (Randsborg et al., 2022). At this point, the concept of rumination plays a critical role in understanding how mental processes are shaped after injury and how anxiety levels increase.

Beyond these physical and behavioral consequences, kinesiophobia is not only a factor that delays return to sport but also negatively influences athletes’ life satisfaction, self-efficacy, and psychological well-being. Previous research has systematically shown that kinesiophobia reduces quality of life and physical activity levels (Luque-Suárez et al., 2019; Vlaeyen & Linton, 2000). Particularly in chronic musculoskeletal conditions, higher levels of kinesiophobia have been associated with poorer overall well-being (Chua et al., 2025). Similarly, among university students, higher levels of kinesiophobia have been found to restrict physical activity and diminish quality of life (Bulguroglu et al., 2023). Thus, kinesiophobia not only creates physical limitations but also directly undermines athletes’ well-being by lowering self-efficacy and life satisfaction.

Furthermore, the psychological consequences of kinesiophobia extend beyond reduced activity levels, as fear of movement undermines athletes’ confidence in their ability to cope with physical and psychological demands successfully. Previous studies have shown that kinesiophobia reduces self-efficacy, crucial for effective rehabilitation and return to play (Clement et al., 2013). This erosion of self-efficacy may contribute to heightened stress, persistent worry about re-injury, and maladaptive cognitive patterns such as rumination, which collectively weaken resilience in the face of adversity (M. Li et al., 2024; Jack et al., 2010). Over time, these psychological challenges compromise athletic performance and threaten career sustainability and long-term mental health (Chua et al., 2025; Bulguroglu et al., 2023).

Taken together, these findings highlight that kinesiophobia is not a unidimensional construct limited to physical avoidance but a multidimensional threat that directly undermines athletes’ well-being. By lowering self-efficacy, life satisfaction, and psychological resilience, kinesiophobia creates a cycle of fear and avoidance that can hinder rehabilitation outcomes and athletes’ broader quality of life (Luque-Suárez et al., 2019; Vlaeyen & Linton, 2000).

### 1.2. Rumination

Rumination is a coping style that emerges after traumatic experiences and leads individuals to become fixated on negative thoughts (Nolen-Hoeksema et al., 2008). This process causes individuals to repetitively and persistently dwell on negative thoughts. Typically focusing on past events, mistakes, or worries, this thinking style is not solution-oriented but instead traps the person in a cycle of mental stagnation (Armutlu, 2019).

The Response Styles Theory, proposed by Nolen-Hoeksema (1991), conceptualizes rumination as a reaction to emotional distress. Nolen-Hoeksema (1991) defined this state as the repetitive mental processing of a stressful event without actively seeking a solution, ultimately drawing the individual into a negative cognitive loop. As a result, being fixated on past negative experiences fosters pessimistic expectations about the future and delays both psychological and physical recovery (Calhoun et al., 2000).

In this context, stress, which lies at the center of the negative mental cycle generated by rumination, is defined as a factor that can adversely affect an individual’s quality of life, hinder the expression of their potential, and make it difficult to adapt to their environment (Hannigan et al., 2004). Especially following traumatic experiences, ruminative thinking patterns can deplete mental resources, making individuals more vulnerable to external stressors (Calhoun et al., 2000). In this regard, rumination increases emotional burden and hampers one’s ability to cope effectively with stress, impairing problem-solving capacity and adaptive functioning (Nolen-Hoeksema et al., 2008).

The adverse effects of rumination are not limited to daily life; they can also directly influence an individual’s professional, academic, and athletic performance. In the sports context, rumination may be triggered when an athlete’s performance falls below the expected target, and this process may continue until the performance gap is reduced or the target is readjusted (Kröhler et al., 2024). Such mental processes can impair the ability to cope with stressors and negatively affect athletes’ performance (Josefsson et al., 2017). These mental patterns, which are frequently observed in athletes during the post-injury period, may hinder individuals from focusing on their physical recovery process and reinforce kinesiophobia by fueling the fear of re-injury. Moreover, meta-analytic findings also support that athletes who adopt effective coping strategies perform better, whereas avoidance-oriented coping strategies may lead to adverse outcomes (Crocker et al., 2015; Nicholls et al., 2016). Considering such cognitive and emotional effects of rumination, understanding how coping styles with stress are shaped is a critical necessity, particularly for enhancing the effectiveness of psychological interventions in athlete rehabilitation processes.

### 1.3. Coping Responses

Stress is a factor that affects every aspect of an individual’s life. When not managed properly, it can negatively impact physical and psychological health and lead to various difficulties in daily life (Yılmaz, 2006). Therefore, developing effective coping responses is essential to minimize the effects of stress and maintain quality of life. Individual characteristics, social conditions, and the challenges encountered shape coping responses. This process involves cognitive and behavioral adaptation mechanisms aimed at recognizing sources of stress, managing them effectively, and maintaining emotional balance (Baltaş & Baltaş, 2018; Ayaz et al., 2020; Sürme, 2019). Appropriate coping responses help individuals regulate their emotions and thoughts, manage difficulties more effectively, and maintain balance in life. When utilized effectively, stress can even become a motivating factor that enhances problem-solving skills (Güçlü, 2001).

One model that offers a structured approach to coping responses is the “Change Accept Let Go Manage Your Lifestyle” (CALM) Model developed by Braham (1998). This model presents coping responses in four stages. The first step, Change, refers to confronting the source of stress and taking active steps to alter the situation. This may include seeking help, time management, setting boundaries, and anticipating stressors. Acceptance emphasizes acknowledging uncontrollable situations without anger and maintaining a positive outlook. Let Go is used to avoid unnecessary stress and develop a balanced perspective. Finally, Manage Your Lifestyle enhances the capacity to cope with stress through exercise, healthy nutrition, relaxation techniques, and emotional support (as cited in Güçlü, 2001). This model supports individuals in managing stress more effectively and aims to preserve the overall quality of life. While coping responses are influenced by personal characteristics, social context, and life challenges, Braham’s CALM model provides a crucial roadmap for establishing a healthier balance and minimizing the adverse effects of stress (Bozhüyük et al., 2012; Yetim & Çevik, 2019).

Coping responses enhance general life satisfaction and play a critical role in athletes’ recovery. In particular, coping strategies directly affect the speed of rehabilitation and the athlete’s motivation to return to sport. Moreover, coping resources such as social support, mindfulness, and self-efficacy have been shown to buffer the adverse effects of stress during rehabilitation and facilitate recovery (Werner et al., 2023). In this context, Braham’s CALM model offers a structured pathway for dealing with such psychological challenges, supporting athletes’ mental well-being and enabling a more efficient and balanced recovery process. In this regard, kinesiophobia and rumination, which commonly develop following injury, create a negative feedback loop that affects both the psychological and physical recovery of athletes. Kinesiophobia, the fear of movement or reinjury, triggers avoidance behaviors and loss of motivation, while a ruminative thought pattern focused on past experiences increases anxiety and hinders recovery. This dynamic may prolong rehabilitation, delay return to sport, and reduce athletic performance. At this point, effective coping responses play a pivotal role in managing symptoms and enhancing psychological flexibility and emotional resilience. Particularly, structured models such as Braham’s CALM approach enable athletes to address external stressors while regulating internal emotional processes. In doing so, athletes can learn to accept uncontrollable circumstances, reduce mental burdens, and actively contribute to recovery through lifestyle adjustments.

Fredrickson’s (2001) Broaden and Build Theory also emphasizes the role of positive emotions in expanding attention and enhancing cognitive resources. Experiencing positive emotions allows individuals to develop more creative and adaptive coping responses in stressful situations, making them more flexible and resilient in the face of challenges. In athletes, encouraging positive emotions during recovery helps reduce psychological distress and contributes to a healthier adjustment process. This reconstruction of mental resources facilitates coping with existing stressors and equips individuals with a more robust attitude toward future uncertainties. All these processes are closely linked to the development of psychological flexibility.

### 1.4. Psychological Flexibility

Psychological flexibility refers to an individual’s ability to adapt to changing life circumstances and act according to personal values. As a core component of mental well-being, it plays a crucial role in coping with stress, managing emotions in a balanced manner, and maintaining engagement in meaningful activities. This skill involves being fully present in the moment, accepting thoughts and emotions as they are, and making decisions aligned with one’s values (Yıldız, 2021; Can & Onnar, 2025).

In other words, psychological flexibility refers to an individual’s ability to align with their values while maintaining awareness of the present moment, without becoming entangled in past or future concerns (Luoma et al., 2017). This flexibility enables individuals to openly, consciously, and nonjudgmentally accept their current experiences, allowing them to adapt to changing circumstances and behave according to their values (Powers et al., 2009). In this way, when faced with challenging situations, the individual can respond more adaptively with mindful awareness and flexible behavior, rather than becoming stuck in rigid thought patterns.

Moreover, psychological flexibility allows individuals to approach their thoughts and emotions with a mindful detachment, seeking to make sense of them rather than trying to suppress or change them. This process supports continued action in line with one’s values and fosters the capacity to consciously reshape behaviors (Kashdan & Rottenberg, 2010). This ability is critical, particularly for athletes navigating the challenging period marked by physical limitations and emotional fluctuations following an injury. Individuals with psychological flexibility can recognize and accept adverse experiences instead of suppressing or denying them, thereby developing the capacity to maintain value-consistent behaviors (S. Kılıç & Kabasakal, 2025). This approach mitigates the impact of kinesiophobia and ruminative thinking patterns, commonly observed in athletes, by promoting a more balanced mental framework. Therefore, psychological flexibility is a buffer against stressful experiences and an adaptive mechanism that facilitates the individual’s active and mindful participation in recovery (Aydoğdu & Karataş, 2025). Especially during the return to sport phase, maintaining motivation despite emotional fluctuations, sustaining adaptive behaviors, and effectively utilizing internal resources rely heavily on psychological flexibility. This process extends beyond physical recovery, contributing to the psychological empowerment of athletes and fostering a holistic approach to healing. In turn, athletes can regain physical and mental strength, enabling a more resilient return to sport.

### 1.5. The Present Study

This study aims to examine how the relationship between kinesiophobia and rumination in athletes is shaped within the framework of the mediating role of coping strategies and psychological flexibility. The proposed model, which aspires to offer an interdisciplinary contribution to the field of sport psychology, is designed to foster a perspective that supports both the acceleration of athletes’ rehabilitation processes and the enhancement of their psychological resilience and performance.

Current research predominantly focuses either on physical recovery processes (Zetaruk et al., 2005; Koz & Ersöz, 2010) or on the individual psychological effects of fear of reinjury (Podlog & Eklund, 2005; Ivarsson & Johnson, 2010; Kayhan et al., 2019), and kinesiophobia after injury has been studied separately, especially in the context of lower extremity injuries (Ardern et al., 2013; Houston et al., 2014; Ardern et al., 2014; Paterno et al., 2018; Theunissen et al., 2020; Fukano et al., 2020; Vascellari et al., 2021; Jedvaj et al., 2021; Castanho et al., 2021; Haraldsdottir & Watson, 2021; Cross et al., 2021; Slagers et al., 2021; Reinking et al., 2022; Raizah et al., 2022; Alshahrani & Reddy, 2022; Watanabe et al., 2023; Ohji et al., 2023; Ambegaonkar et al., 2024). Rumination, on the other hand, has been separately discussed in the context of depression, anxiety, psychosis, insomnia, suicidal tendencies, and cognitive control disorders (Watkins & Roberts, 2020; Nolen-Hoeksema et al., 2008; J. M. Smith & Alloy, 2009; Kirkegaard Thomsen, 2006; Treynor et al., 2003; Morrison & O’Connor, 2008; Hannigan et al., 2004; Nolen-Hoeksema & Jackson, 2001; Berry et al., 2005; Ward et al., 2003; Sansone & Sansone, 2012). Moreover, while coping strategies in athletes have been examined in terms of individual, environmental, and contextual factors (Anshel et al., 2000; Anshel & Anderson, 2002; Nicholls & Polman, 2007; Crocker et al., 2015; Kaiseler et al., 2009; Kerdijk et al., 2016; Crocker et al., 2017; Cosma et al., 2020; Doron & Martinent, 2021), their interactive role with kinesiophobia and rumination has not been explored in detail. Similarly, psychological flexibility has been investigated with regard to performance in team sports, burnout, rehabilitation adherence, sport-specific flexibility assessments, mindfulness-based approaches, flexibility and well-being, physical activity continuity, and occupational burnout (Johles et al., 2020; Chang et al., 2018; DeGaetano et al., 2016; Swettenham & Whitehead, 2022; Carrança et al., 2019; Mooney, 2022; Jenkins et al., 2019; Ruiz & Odriozola González, 2017; Ronkainen et al., 2024). However, no comprehensive and systematic model has yet been proposed that integrates these four constructs (kinesiophobia, rumination, coping strategies, and psychological flexibility) in a multivariate and interactive framework.

In this context, the most significant scientific and innovative aspect of this study lies in its holistic modeling of psychological vulnerabilities in athletes not merely as individual reactions, but as a multidimensional structure, aiming to understand how kinesiophobia and rumination are shaped by coping strategies and psychological flexibility capacity. Furthermore, this model highlights the determinative role of psychological factors in rehabilitation, aiming to support physical recovery after injury and enhance athletes’ psychological resilience and performance sustainability.

This study proposes an innovative and theoretically grounded four-dimensional model comprising kinesiophobia, rumination, coping responses, and psychological flexibility to explain and manage the psychological reactions that emerge in athletes following injury. The proposed model addresses a significant theoretical gap in the literature by drawing upon Braham’s Dynamic Constraint Coping Response (DCCR) Model, Lethem’s Fear Avoidance Model, and Fredrickson’s Broaden and Build Theory. While existing research tends to examine these variables either independently or in dyadic relationships, this study is the first to integrate all four variables within a holistic, interactive, and process-oriented framework. The model presents a unique structure that explains the relationship between kinesiophobia and rumination in athletes, while highlighting the mediating roles of coping responses and psychological flexibility. In this respect, it diverges from traditional rehabilitation approaches that focus solely on physical recovery, emphasizing instead the interactive nature of psychological processes. This multidimensional perspective aims to enhance return to play speed, psychological well-being, and performance sustainability. Furthermore, the model enables coaches, sport psychologists, and other practitioners to develop individualized, awareness-based assessment and intervention strategies, thereby contributing to more comprehensive and targeted support for athletes’ psychological processes. In conclusion, this study integrates sport psychology, clinical psychology, and sport sciences through an interdisciplinary approach, contributing an original theoretical framework and an innovative practice-oriented model to the literature; most importantly, demonstrating its mediating effect provides a fundamental reference point for future research.

**H1.**  *Kinesiophobia that develops in athletes after injury increases rumination levels.*

**H2.**  *Coping skills have a mediating role; these skills weaken the relationship between kinesiophobia and rumination.*

**H3.**  *Psychological flexibility has a mediating role; this variable reduces the relationship between kinesiophobia and rumination.*

**H4.**  *Coping skills and psychological flexibility have a mediating role; these two variables decrease the overall effect of kinesiophobia on rumination.*

## 2. Materials and Methods

### 2.1. Research Model

Within the scope of this research, a correlational survey model was employed to identify the relationships between variables. This model examines how two or more variables change together and aims to determine the relationships’ significance and strength. As a non-experimental method, it provides descriptive information about the direction and strength of the relationships, thereby formulating possible predictions (Karasar, 2016; Christensen et al., 2010).

### 2.2. Determination of Sample Size

An a priori power analysis was conducted for a fixed linear multiple regression (F-test) model to determine the required sample size for detecting a small effect size (f^2^ = 0.05) with two predictors. The significance level (α) was 0.05, and the desired statistical power (1 − β) was 0.95. The analysis indicated that a minimum sample size of 312 participants is required to achieve the specified power. The noncentrality parameter (λ) was calculated as 17.4, with a critical F value of 2.63. The degrees of freedom for the numerator and denominator were 2 and 309, respectively. The actual power achieved with this sample size is 0.950, confirming that the study design is adequately powered to detect the hypothesized effect.

### 2.3. Population and Sample

The study population consists of all individuals targeted by the research and considered in the sample selection process (Çıngı, 1994; Şimşek, 2015). This study’s population includes licensed athletes actively participating in sports clubs across Türkiye. In Türkiye, a licensed athlete refers to individuals who are active in a specific sport discipline and registered in the relevant federation’s official system, through which they are granted an athlete license. This license certifies that the athlete meets the required health and administrative conditions and demonstrates their eligibility to participate in official competitions organized under the supervision of the federation (Gençlik ve Spor Bakanlığı, 2019).

The sample group was determined using the simple random sampling method. This method ensures that every individual in the population has an equal chance of being included in the sample, thereby increasing its representativeness (Çıngı, 1994). Accordingly, online survey forms were distributed to various sports clubs throughout Turkey, and participants who met the inclusion criteria and volunteered to participate were selected for the sample.

The inclusion criteria for participation in the study were as follows:

- Being 18 years of age or older,
- Having been a licensed athlete for at least 3 years,
- Having experienced an injury related to the lower or upper extremities,
- Having been away from the field for at least 3 months due to that injury,
- Returning to the field post-injury and continuing to compete at a competitive level.

The study sample was the 390 athletes who met these criteria and voluntarily agreed to participate.

All participants in this study were athletes who had previously sustained a sports-related injury that resulted in a minimum of three months of absence from training and competition. Importantly, inclusion criteria required that all athletes had successfully returned to their sport and actively competed during data collection. Thus, none of the participants were in the acute injury phase or undergoing rehabilitation; instead, they were in the post-injury active phase, fully reintegrated into training and competition. This criterion ensured that the study sample represented athletes with direct lived experience of injury and recovery, while simultaneously allowing for the examination of the long-term psychological consequences of kinesiophobia beyond the immediate rehabilitation process. Clarifying this point is crucial, as it underscores that participants’ responses reflect the perspective of athletes who had already returned to sport, thereby reducing ambiguity regarding whether they were injured, recovering, or recalling past injury.

During the data collection, the instruments prepared via Google Forms were sent online to sports clubs operating in various provinces across Turkey. Participation in the study was voluntary and limited to individuals who met the predefined inclusion criteria. In total, 390 athletes from different sports branches and clubs participated in the study. This number is considered sufficient to ensure the reliability of the research sample size.

### 2.4. Study Model

This study uses a correlational survey model to examine the relationship between kinesiophobia and rumination in athletes and the mediating role of coping responses and psychological flexibility in this relationship. Kinesiophobia is a fear triggered by a perceived threat of movement, and it may activate rumination, which involves repetitive negative thoughts. The study aims to reveal how coping responses and psychological flexibility mediate this relationship.

Mediation analysis is a robust statistical approach that allows for exploring underlying processes in the relationship between two variables (Gürbüz, 2019). In this context, the mediator variable (M) acts as a bridge that explains the effect of the independent variable (X) on the dependent variable (Y) (Baron & Kenny, 1986). In this study, the relationship between kinesiophobia (X) and rumination (Y) was analyzed through the mediating roles of coping responses (M1) and psychological flexibility (M2).

**Figure 1:**
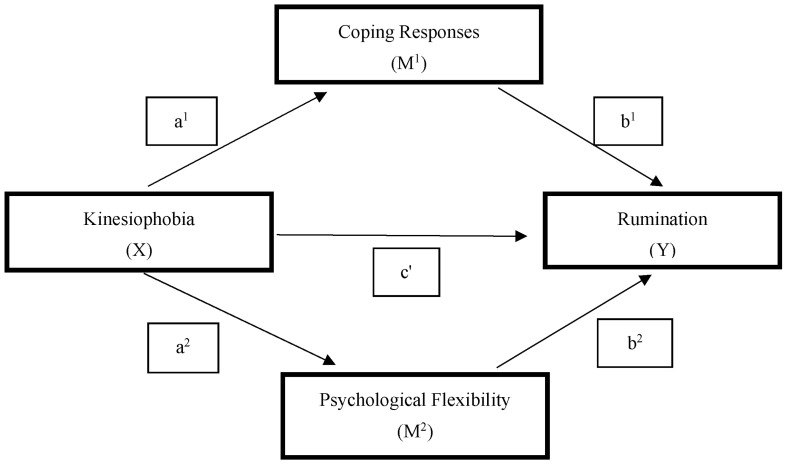
Mediation effect model of psychological flexibility and coping responses.

Figure 1 presents the mediation model showing the indirect effects of psychological flexibility and coping responses.

### 2.5. Data Collection Form

#### 2.5.1. Personal Information Form

As part of the study, a personal information form consisting of six variables was prepared to collect demographic data regarding the participants. The variables included in this form were: gender, age, years of athletic experience, type of sport, type of previous injury, and duration.

#### 2.5.2. Tampa Scale for Kinesiophobia (TSK)

The original version of the Tampa Scale for Kinesiophobia (TSK) was developed by Miller et al. in 1991 (Miller et al., 1991), and its final form was published by Vlaeyen et al. (1995). The Turkish adaptation and cultural validation of the scale were conducted by Tunca Yılmaz et al. (2011). The TSK is a checklist consisting of 17 items (e.g., “I am afraid that I might injure myself if I exercise”) and is widely used in clinical conditions such as acute/chronic low back pain, fibromyalgia, musculoskeletal injuries, and whiplash syndrome.

The scale is structured as a 4-point Likert-type instrument (1 = Strongly Disagree, 4 = Strongly Agree). Items 4, 8, 12, and 16 are reverse-scored, and the total score is obtained by summing all item scores, ranging from 17 to 68. A higher total score indicates a higher level of kinesiophobia. The test-retest reliability of the scale was calculated as 0.806 (95% CI = 0.720–0.867) (Tunca Yılmaz et al., 2011). This study evaluated the scale’s internal consistency using Cronbach’s alpha coefficient, which was calculated as 0.77.

#### 2.5.3. Sport Competition Rumination Scale

The Sport Competition Rumination Scale was developed by Michel Kröhler, Krys, and Berti (Michel-Kröhler et al., 2021), and its Turkish adaptation was carried out by Karafil and Pehlivan (2023). This scale is used to identify rumination patterns, repetitive cognitive processes that may negatively affect performance experienced by athletes during sports competitions (Karafil & Pehlivan, 2023).

The scale consists of six items, has a single factor structure, and is administered using a five-point Likert scale (1 = Strongly Disagree, 5 = Strongly Agree). An example item is: “I often reevaluate my memories related to unsuccessful situations in competitions.” In the reliability study of the Turkish adapted form, the Cronbach’s alpha internal consistency coefficient was found to be 0.87. In the confirmatory factor analysis of the scale, the factor loadings of each item ranged between 0.555 and 0.861. (Karafil & Pehlivan, 2023). In the present study, the scale’s internal consistency was reassessed, and the Cronbach’s alpha coefficient was found to be 0.88.

#### 2.5.4. Coping Response Inventory

The Ways of Coping Scale was initially developed by Moos (1993), and its Turkish adaptation, validity, and reliability study was conducted by Ballı and Kılıç (2016). The present study utilized only the subscale related to coping responses, specifically the four factors of logical analysis, positive appraisal, seeking guidance and support, and problem solving.

This section, consisting of 24 items (e.g., “I think of different ways to cope with problems”), is administered using a 5-point Likert-type rating system. Participants rate the statements on a scale from “1 = Never” to “5 = Always.” In the original studies, the internal consistency coefficient of the scale was reported as 0.93. In the confirmatory factor analysis, the factor loadings of each item were found to range between 0.65 and 0.89 (Ballı & Kılıç, 2016). In this study, the reliability of the form used was evaluated with Cronbach’s alpha coefficient and calculated as 0.94.

#### 2.5.5. Psychological Flexibility Scale

The Psychological Flexibility Scale was developed by Francis et al. (2016), and its Turkish adaptation, validity, and reliability study was conducted by Karakuş and Akbay (2020). The scale consists of 28 items and includes five subdimensions: values-based action, present moment awareness, acceptance, self as context, and cognitive defusion. An example item is: “I know what is important to me and where I want to be in my life.”

Items are rated on a 7-point Likert scale, ranging from 1 (Strongly Disagree) to 7 (Strongly Agree). The total score ranges between 28 and 196. Items 2, 3, 5, 6, 8, 18, 20, 22, 23, 24, and 25 are reverse-scored to control for negatively worded statements. Higher scores on each subdimension indicate a higher level of psychological flexibility. The Cronbach’s alpha coefficient for the Turkish adaptation was reported as 0.79. The factor loadings of the scale range between 0.47 and 0.81 (Karakuş & Akbay, 2020). In the present study, the scale’s internal consistency was calculated using Cronbach’s alpha, yielding a coefficient of 0.88.

Table 1 presents the distributions of the main demographic variables of the athletes who participated in the study.

Table 2 shows that all the scales used in the study demonstrated high internal consistency. The Cronbach’s alpha coefficient was calculated as 0.770 for the Kinesiophobia Scale, 0.884 for the Rumination Scale, 0.886 for the Psychological Flexibility Scale, and 0.941 for the Coping with Stress Scale. These findings indicate that the participants’ responses were consistent across the items and that each scale can be considered a reliable measurement tool.

According to psychometric evaluation criteria, Cronbach’s alpha values of 0.70 or above indicate an acceptable level of internal consistency (George & Mallery, 2003).

As shown in Table 3, all scales demonstrated acceptable levels of model fit. For the Kinesiophobia scale, the χ^2^/df value (2.13) indicated good fit, while the CFI (0.92) and TLI (0.92) reached the acceptable threshold. RMSEA (0.053) and SRMR (0.054) values were also acceptable. The Rumination scale showed excellent fit, with χ^2^/df (1.43) well below 3, and both CFI (0.99) and TLI (0.99) exceeding the 0.95 criterion. RMSEA (0.033) and SRMR (0.015) further supported strong model fit. For the Psychological Flexibility scale, χ^2^/df (2.70) indicated acceptable fit, with CFI (0.95) and TLI (0.95) reaching the good fit threshold. RMSEA (0.055) and SRMR (0.048) were within the range of good fit. The Coping with Stress scale demonstrated strong fit, with χ^2^/df (2.50) below 3, CFI (0.96) and TLI (0.96) exceeding 0.95, and RMSEA (0.049) and SRMR (0.042) indicating perfect fit. Overall, the Rumination scale displayed the strongest fit indices, while the remaining scales ranged from acceptable to reasonable levels of fit, supporting the measurement validity of the models (Hu & Bentler, 1999; Kline, 2016).

### 2.6. Data Analysis

The data analysis phase utilized IBM SPSS Statistics 26.0 and PROCESS Macro 4.0. Descriptive statistics were calculated, including percentages (%) and frequency distributions. In addition, an a priori power analysis was conducted using G*Power software (v 3.1) to assess the statistical adequacy of the sample size. Pearson correlation analysis was employed to examine the relationships among variables, and Fisher’s Z transformation test was conducted to compare the strength and direction of these correlations. A linear regression analysis was conducted to examine the predictive effect of kinesiophobia on rumination.

Moreover, mediation analysis (Model 4) using the PROCESS Macro was performed to test the mediating roles of psychological flexibility and coping responses in the relationship between kinesiophobia and rumination. To determine the statistical significance of indirect effects, the bootstrap method was employed with 5000 resamples, and 95% confidence intervals were calculated (Bias Corrected and Accelerated—BCa CI). The mediation effect was considered statistically significant if the confidence interval did not include zero (Gürbüz, 2019; Hayes, 2013).

## 3. Results

Table 4 shows a positive. There is a significant correlation between the kinesiophobia and rumination scales (r = 0.303, *p* < 0.001). Similarly, significant but weaker correlations were found between the kinesiophobia scale and the psychological flexibility scale (r = 0.175, *p* < 0.001), as well as the coping responses scale (r = 0.181, *p* < 0.001).

A positive and significant correlation was also observed between the rumination scale and the psychological flexibility scale (r = 0.274, *p* < 0.001), and between the rumination scale and the coping responses scale (r = 0.285, *p* < 0.001). The correlation between psychological flexibility and coping responses was moderate and statistically significant (r = 0.463, *p* < 0.001).

Furthermore, Fisher’s Z transformations were calculated to allow a more straightforward interpretation of the strength and direction of these relationships. The correlation coefficient between kinesiophobia and rumination (r = 0.303) had a Fisher Z transformation of z’ = 0.309. For the correlation between kinesiophobia and psychological flexibility (r = 0.175), the z-score was 0.171; for the correlation between kinesiophobia and coping responses (r = 0.181), the z was 0.182. The Fisher Z transformation for the correlation between rumination and psychological flexibility (r = 0.274) was z’ = 0.276; for rumination and coping responses (r = 0.285), z’ = 0.287; and for psychological flexibility and coping responses (r = 0.463), z’ = 0.497.

As seen in Table 5, the regression model constructed to examine the effect of kinesiophobia on rumination levels among participants was found to be statistically significant (F_1,419_ = 39.082, *p* < 0.001). According to the t-test results for the significance of the regression coefficient, kinesiophobia significantly predicted rumination (β = 0.223, t = 6.254, *p* < 0.001). According to this model, kinesiophobia explained 9.2% of the variance in participants’ rumination levels (R^2^ = 0.092).

**Figure 2:**
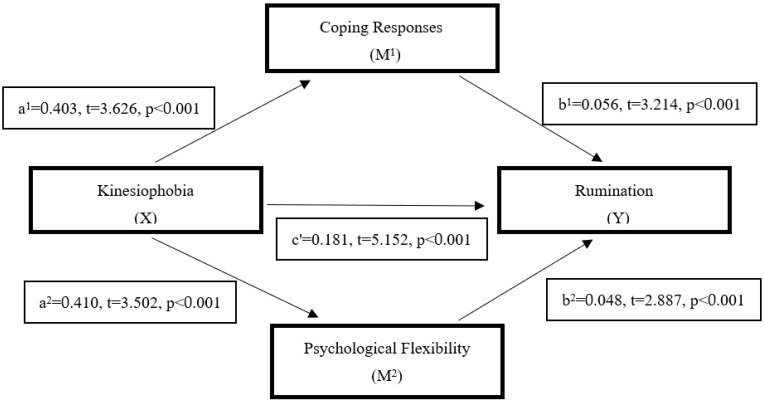
The mediating role of psychological flexibility and coping responses in the relationship between kinesiophobia and rumination. Note: total effect = 0.223, *p* < 0.001; direct effect = 0.181, *p* < 0.001; indirect effect = 0.042, 95% CI [0.017, 0.072].

Finally, when Figure 2 was examined, the direct effect of kinesiophobia on coping responses (a1 = 0.403, t = 3.626, *p* < 0.001) and psychological flexibility (a2 = 0.410, t = 3.502, *p* < 0.001) was significant. In addition, the direct effects of coping responses (b1 = 0.056, t = 3.214, *p* < 0.001) and psychological flexibility (b2 = 0.048, t = 2.887, *p* < 0.001) on rumination were significant. When kinesiophobia, psychological flexibility, and coping responses were included in the model simultaneously, the relationship between kinesiophobia and rumination decreased in terms of direct effect. However, the significance value remained at the same level (c’ = 0.181, t = 5.151, *p* < 0.01). The values of the effects between the variables of the model tested in the research are presented in Table 6.

As shown in Table 6, the total indirect effect of kinesiophobia on rumination through psychological flexibility and coping responses was statistically significant (point estimate = 0.057, 95% BCa CI [0.025–0.096]).

Examining the effects of the mediating variables in the model, both psychological flexibility (point estimate = 0.027, 95% BCa CI [0.005–0.053]) and coping responses (point estimate = 0.031, 95% BCa CI [0.006–0.064]) had statistically significant total indirect effects on rumination.

Furthermore, the comparison of the relative strength of the mediators was not statistically significant (point estimate = −0.004, 95% BCa CI [−0.048–0.033]), indicating that coping responses had a slightly more substantial mediating effect (point estimate = 0.004).

## 4. Discussion

The results revealed statistically significant relationships among kinesiophobia, rumination, psychological flexibility, and coping responses. A positive and significant correlation was found between kinesiophobia and rumination (r = 0.303, *p* < 0.001). This finding suggests that fear related to physical movement may lead individuals to engage more frequently in negative thought cycles. Given that rumination is characterized by the repetitive processing of thoughts, especially those related to pain and physical discomfort, this pattern may play a role in maintaining or exacerbating kinesiophobia. Similarly, M. Li et al. (2024) reported a significant positive correlation between rumination and kinesiophobia in their study. Larsson et al. (2016) found that kinesiophobia was significantly associated with pain intensity and self-perceived health. This finding suggests that avoidant reactions to physical strain are connected to individuals’ pain perception and general health evaluations.

The findings of this study further suggest that kinesiophobia is not only related to increased rumination but may also directly undermine athletes’ well-being. Literature has demonstrated that kinesiophobia weakens self-efficacy, decreases life satisfaction, and impairs psychological health (Clement et al., 2013; Jack et al., 2010). For example, in fibromyalgia patients, high kinesiophobia has been associated with lower self-efficacy and reduced physical activity levels. Likewise, in individuals with osteoarthritis, elevated kinesiophobia scores were significantly related to poorer quality of life outcomes (Chua et al., 2025). Recent studies also emphasize that kinesiophobia restricts participation in physical activity and reduces well-being among university students (Bulguroglu et al., 2023), while systematic reviews indicate that it significantly diminishes quality of life in people with chronic musculoskeletal pain (Luque-Suárez et al., 2019). Therefore, kinesiophobia should be considered a multidimensional threat to athletes’ well-being, not only delaying return to play but also impairing their broader psychological functioning, career sustainability, and psychological resilience.

Significant yet weaker positive correlations were also found between kinesiophobia and psychological flexibility (r = 0.175, *p* < 0.001), and between kinesiophobia and coping responses (r = 0.181, *p* < 0.001). It is known that individuals with higher levels of psychological flexibility can more effectively manage life challenges. In this context, higher levels of kinesiophobia in individuals with low psychological flexibility may be explained by the development of rigid and avoidant responses toward pain or physical exertion. Similarly, the relationship with coping responses can be interpreted to mean that individuals’ behavioral and cognitive strategies in response to stress may influence their fear of movement (Nasir et al., 2023). Harris (2016) noted that individuals with high psychological flexibility are open to negative internal experiences and behave in ways consistent with their values by accepting these experiences. In contrast, individuals with low psychological flexibility tend to perceive negative emotions and thoughts as overwhelming and challenging to manage.

Significant and positive correlations were also found between rumination and psychological flexibility (r = 0.274, *p* < 0.001), as well as between rumination and coping responses (r = 0.285, *p* < 0.001). It can be inferred that a ruminative thinking style may weaken psychological flexibility and reduce one’s capacity to manage stress, leading individuals to adopt less functional coping responses. In this regard, the moderate and significant relationship found between psychological flexibility and coping responses (r = 0.463, *p* < 0.001) suggests that individuals with higher psychological flexibility are more likely to develop adaptive cognitive and emotional strategies to cope with stress (Bonanno & Burton, 2013; Uludağ, 2021). Indeed, a study conducted by Sarımen Ündar and Canpolat (2025), among university students, demonstrated that psychological flexibility was positively associated with certain adaptive coping styles, while negatively associated with avoidant strategies (Watkins & Roberts, 2020; Blanke et al., 2022; Kato, 2020; Krys & Reininger, 2025).

According to the findings, kinesiophobia was found to significantly predict rumination (β = 0.223, *p* < 0.001). This suggests that as individuals’ fear of movement increases, they may be more likely to experience repetitive and negative thought patterns. In other words, kinesiophobia may increase rumination by causing individuals to mentally process uncertainty and anxiety following an injury. The model accounts for 9.2% of the variance in rumination levels (R^2^ = 0.092), indicating a statistically significant, albeit low, predictive effect. These findings demonstrate that kinesiophobia is not merely a physical limitation but also a cognitive factor that affects athletes’ psychological processes. In this context, athletes with high levels of kinesiophobia may engage in more frequent self-reflection during the post-injury period, mentally replay their experiences more often, and as a result, may exhibit reduced psychological flexibility.

In a study by Acar (2022), psychological flexibility explained 16% of the variance in coping responses. Similarly, in Sarımen Ündar and Canpolat’s (2025) research, coping response subdimensions significantly predicted psychological flexibility. These findings suggest that psychological flexibility plays a decisive role in individuals’ ability to manage stress and that the relationship between psychological flexibility and coping responses is strong (Blanke et al., 2022; Wersebe et al., 2018).

According to the mediation analysis, which constitutes the study’s primary aim, psychological flexibility and coping responses play statistically significant mediating roles in the effect of kinesiophobia on rumination. In the overall model, the direct effect of kinesiophobia on rumination was significant (β = 0.223, *p* < 0.001). However, when psychological flexibility and coping responses were included, the strength of this relationship decreased (β = 0.181, *p* < 0.001) while remaining statistically significant. This indicates that the mediating variables exert a partial mediating effect on the relationship. When the mediators were examined separately, both psychological flexibility (point estimate = 0.027, 95% CI [−0.005–−0.053]) and coping responses (point estimate = 0.031, 95% CI [0.006–0.064]) demonstrated significant mediation effects in the relationship between kinesiophobia and rumination. This finding suggests that individuals’ tendencies to avoid physical movement are directly associated with negative thought patterns and influenced by how they cognitively evaluate the situation and manage stress through coping responses.

The current findings align with prior research examining the association between kinesiophobia and rumination. Specifically, studies have shown that catastrophic thoughts related to pain increase avoidance behaviors, thereby elevating kinesiophobia, a process that is closely linked to rumination (Craner et al., 2016; Duport et al., 2022). Catastrophizing involves exaggerating threats and excessively focusing on adverse outcomes, which leads individuals to perceive pain as more intense and threatening. This, in turn, creates a vicious cycle of avoidance and physical inactivity (Caneiro et al., 2021; Szabo et al., 2022; Karabay et al., 2023).

It has been emphasized that rumination depletes cognitive resources and chronically activates physiological stress responses, thereby negatively impacting individuals’ overall health status (Szabo et al., 2022; Kucyi et al., 2014). The association between pain-related rumination and higher pain perception, as well as poorer clinical outcomes in individuals with chronic pain (Karabay et al., 2023), supports the significant relationship found in our study between rumination and kinesiophobia. Particularly, the emotional distress and negative attitudes toward pain that intensify with ruminative thinking may reinforce fear and avoidance behaviors related to physical activity.

Psychological flexibility allows individuals to align with their values despite distressing internal experiences. At the same time, effective coping responses facilitate more adaptive management of stressors and help reduce repetitive cognitive processes. Indeed, the literature suggests that both psychological flexibility and coping responses serve as protective factors against cycles of rumination (Kashdan & Rottenberg, 2010; Bonanno & Burton, 2013).

In a recent study, M. Li et al. (2024) found that rumination and psychological resilience sequentially mediated the relationship between symptom burden and kinesiophobia in patients with chronic heart failure (CHF).

Similarly, a mediation analysis conducted by Rueda and Valls (2020) revealed that when individuals develop context-appropriate coping responses to stress, psychological flexibility significantly mediates this process. Their findings indicate that psychological flexibility meaningfully influences the relationship between stress coping abilities and outcome variables, enhancing individuals’ adaptive capacity in the face of environmental demands.

When comparing the mediating effects, coping responses appear to have a more substantial indirect effect than psychological flexibility. This suggests that coping responses may play a more functional role than psychological flexibility in preventing cognitive strain induced by kinesiophobia from evolving into ruminative thought patterns. Especially because coping responses enable individuals to reappraise situations, act with a problem-solving orientation, and utilize support resources effectively, they may exert a protective influence against mental repetitions and negative thought cycles.

The literature also demonstrates that psychological flexibility enables individuals to accept negative internal experiences without judgment, focus on the present, and engage in value-driven behaviors, thereby mitigating the impact of rumination (Kashdan & Rottenberg, 2010; Francis et al., 2016; Armutlu, 2019; Karakuş & Akbay, 2020). However, current findings suggest that coping responses are more prominent in this process. This indicates that how individuals evaluate and respond to environmental stressors plays a central role in influencing ruminative tendencies (Ballı & Kılıç, 2016; Sürme, 2019).

## 5. Limitations

This study has several limitations that should be acknowledged. First, the research was conducted only with licensed athletes who had previously experienced an injury and were still actively participating in sports. This restricts the generalizability of the findings to other populations, such as recreational athletes, non-athletes, or individuals with chronic health conditions. Therefore, caution should be exercised when applying these results to broader groups (Creswell & Creswell, 2018).

Second, the cross-sectional design precludes establishing causal inferences among the examined variables. Although significant associations were found between kinesiophobia, rumination, psychological flexibility, and coping responses, the directionality of these relationships cannot be conclusively determined. Longitudinal or experimental studies are required to clarify whether kinesiophobia leads to increased rumination and reduced psychological well-being, or whether these psychological processes reciprocally reinforce fear of movement (Synnott et al., 2015; Vlaeyen & Linton, 2012).

Third, the data relied exclusively on self-report instruments (Personal Information Form, Tampa Scale of Kinesiophobia, Sport Competition Rumination Scale, and Coping with Stress Scale). While self-report measures are widely used in psychological and behavioral research, they are subject to several biases, including social desirability and recall bias. Participants’ tendency to evaluate themselves more positively and the subjectivity of their responses may have influenced the results. In future studies, incorporating objective indicators, such as clinical assessments, behavioral observations, or physiological markers, would provide a more comprehensive and valid evaluation (Podsakoff et al., 2003).

In addition, only a limited set of demographic variables, such as gender, age, years of sports experience, type of sport, type of previous injury, and duration of injury, were examined. Other potentially relevant sociodemographic, psychological, or contextual factors (e.g., socioeconomic status, training intensity, access to psychological support) were not included. As a result, the explanatory scope of the study remains limited (A. M. Smith et al., 2020).

Finally, the study did not control for confounding variables such as injury severity, recovery duration, or prior psychological treatment, which may have influenced the observed relationships. Future research should employ experimental and longitudinal designs while considering broader psychosocial and contextual variables to obtain more reliable and comprehensive results (Ardern et al., 2016). By openly acknowledging these limitations, the present study strengthens the credibility of its findings and highlights the need for further investigation into the complex interplay among kinesiophobia, rumination, psychological flexibility, and coping responses in athletes.

## 6. Recommendations

### 6.1. Recommendations for Future Research

Future studies could benefit from qualitative methods such as interviews or focus groups to better understand kinesiophobia, rumination, and psychological flexibility among athletes. Comparative research across different sports, particularly between individual and team athletes, may also shed light on variations in psychological processes. In addition, longitudinal studies examining the impact of coping strategies on psychological well-being during the return to sport phase would provide more unmistakable evidence of causal relationships.

### 6.2. Applicable Suggestions

The findings emphasize the importance of interventions that enhance psychological flexibility and strengthen coping skills in athletes recovering from injury. In this context, group-based psychoeducational programs within sports clubs could be implemented, and in-service training for sports psychologists and physiotherapists may improve the quality of interventions. Moreover, multidisciplinary teams monitoring athletes’ physical and psychological recovery could be key in reducing rumination and fostering resilience throughout rehabilitation.

## 7. Conclusions

This study highlights the crucial mediating role of psychological flexibility and coping strategies in the relationship between kinesiophobia and rumination among athletes. In particular, coping strategies’ more substantial protective effect points to a new focus for mental health interventions. The findings suggest that enhancing athletes’ internal awareness and solution-focused and support-seeking coping skills can foster psychological resilience. Such an approach may reduce ruminative thinking and support a sustainable return to performance after injury.

## Figures and Tables

**Table 1:** Descriptive statistics of the participants.

*N* = 390	Groups	Frequency	Percentage (%)
**Gender**	Female	225	57.7
	Male	165	42.3
**Age**	18–23	131	33.6
	24–29	135	34.6
	30–35	124	31.8
**Sports branch**	Football	113	29.0
	Basketball	75	19.2
	Boxing	36	9.2
	Wrestle	47	12.1
	Athletics	57	14.6
	Handball	62	15.9
**Years of experience in sport**	3–8	101	25.9
	9–14	138	35.4
	15+	151	38.7
**Sport type**	Individual	140	35.9
	Team	250	64.1
**Injury type**	Upper extremity	193	49.5
	Lower extremity	197	50.5
**Injury period**	179 days	166	42.6
	181–365	122	31.3
	366 days +	102	26.2

**Table 2:** Reliability analysis results of the scales.

Scale	Construct	Number of Items	Cronbach’s Alpha
Tampa Scale for Kinesiophobia	kinesiophobia	17	0.770
Sport Competition Rumination Scale	rumination	6	0.884
Coping Response Inventory	psychological flexibility	28	0.886
Psychological Flexibility Scale	coping responses	24	0.941

**Table 3:** Fit Indices of the Measurement Models.

Scale	Construct	χ^2^/df	CFI	TLI	RMSEA	SRMR
Tampa Scale for Kinesiophobia	kinesiophobia	2.13	0.92	0.92	0.053	0.054
Sport Competition Rumination Scale	rumination	1.43	0.99	0.99	0.033	0.015
Coping Response Inventory	psychological flexibility	2.70	0.95	0.95	0.055	0.048
Psychological Flexibility Scale	coping responses	2.50	0.96	0.96	0.049	0.042

**Table 4:** Descriptive statistics and Pearson correlation coefficients for the correlations between the variables.

Variables	1	2	3	4
1- Kinesiophobia	-			
2- Rumination	0.303 **	-		
3- Psychological flexibility	0.175 **	0.274 **	-	
4- Coping responses	0.181 **	0.285 **	0.463 **	-
Min.	17.00	6.00	76.00	24.00
Max.	68.00	30.00	196.00	120.00
X	39.051	20.707	132.746	93.321
SD	7.853	5.800	18.409	17.494

N = 390, ** *p* < 0.001, Min. = Minimum, Max. = Maximum, X = Mean, SD = Standard Deviation 1-Kinesiophobia, 2- Rumination, 3- Psychological Flexibility, 4- Coping Responses.

**Table 5:** The effect of participants’ kinesiophobia on rumination.

Variables									
Independent	Depend	β	SHB	t	*p*	R	R^2^	F	*p*
**Kinesiophobia**	**Rumination**	0.223	0.036	6.254	<0.001	0.303	0.092	39.082	<0.001

**Table 6:** Indirect effects of kinesiophobia on rumination via psychological flexibility and coping responses.

Scale			Bootstrapping 95% BCa		
	Effects	Point Estimate	SE	Lower	Upper
Tampa Scale for Kinesiophobia	Total Indirect Effects	0.057	0.018	0.025	0.096
Sport Competition Rumination Scale	Psychological Flexibility	0.027	0.012	0.005	0.053
Coping Response Inventory	Coping responses	0.031	0.015	0.006	0.064
Psychological Flexibility Scale	Comparisons				
	C1	−0.004	0.021	−0.048	0.033

N = 390, k = 5000, BCa: Bias Corrected and Accelerated, C1 = Psychological flexibility coping responses.

## Data Availability

The original contributions presented in this study are included in the article. Further inquiries can be directed to the corresponding author.
